# Supplementary material for: The effect of intrapartum prolonged oxygen exposure on fetal metabolic status: secondary analysis from a randomized controlled trial
Source: Front Endocrinol (Lausanne). 2023 Jun 27;14:1204956. doi: 10.3389/fendo.2023.1204956 (PMC10335765; doi:10.3389/fendo.2023.1204956)
Supplement: Supplementary file 3 [file DataSheet_3.zip › Result-X101SC21092977-Z01-J001-B1-42/4.MetDiffAnalysis/O.vs.A/O.vs.A_neg_heatmap.pdf]

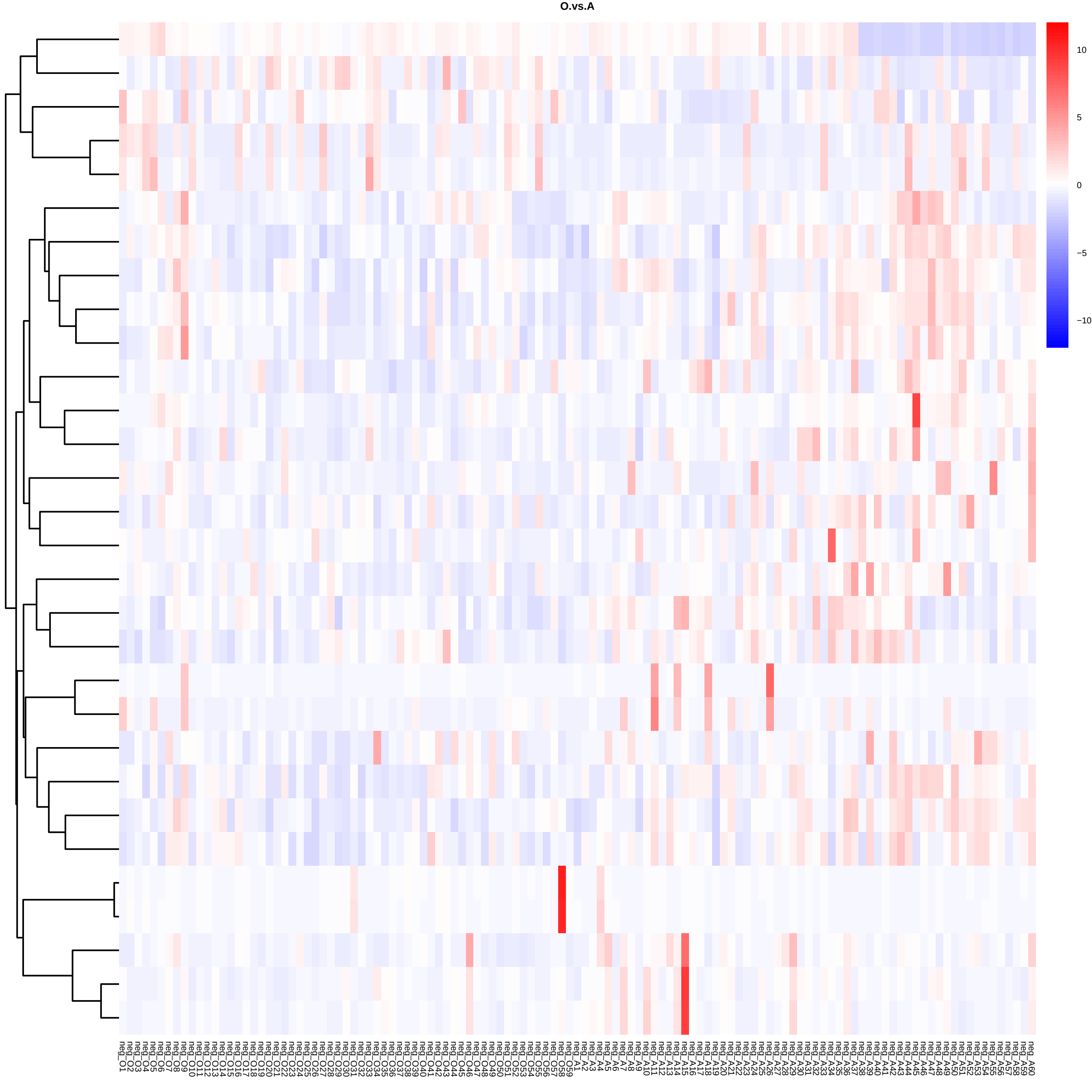
O.vs.A
10
5
0
−5
−10
neg_O1 neg_O2 neg_O3 neg_O4 neg_O5 neg_O6 neg_O7 neg_O8 neg_O9 neg_O10 neg_O11 neg_O12 neg_O13 neg_O14 neg_O15 neg_O16 neg_O17 neg_O18 neg_O19 neg_O20 neg_O21 neg_O22 neg_O23 neg_O24 neg_O25 neg_O26 neg_O27 neg_O28 neg_O29 neg_O30 neg_O31 neg_O32 neg_O33 neg_O34 neg_O35 neg_O36 neg_O37 neg_O38 neg_O39 neg_O40 neg_O41 neg_O42 neg_O43 neg_O44 neg_O45 neg_O46 neg_O47 neg_O48 neg_O49 neg_O50 neg_O51 neg_O52 neg_O53 neg_O54 neg_O55 neg_O56 neg_O57 neg_O58 neg_O59 neg_O60
neg_A1 neg_A2 neg_A3 neg_A4 neg_A5 neg_A6 neg_A7 neg_A8 neg_A9 neg_A10 neg_A11 neg_A12 neg_A13 neg_A14 neg_A15 neg_A16 neg_A17 neg_A18 neg_A19 neg_A20 neg_A21 neg_A22 neg_A23 neg_A24 neg_A25 neg_A26 neg_A27 neg_A28 neg_A29 neg_A30 neg_A31 neg_A32 neg_A33 neg_A34 neg_A35 neg_A36 neg_A37 neg_A38 neg_A39 neg_A40 neg_A41 neg_A42 neg_A43 neg_A44 neg_A45 neg_A46 neg_A47 neg_A48 neg_A49 neg_A50 neg_A51 neg_A52 neg_A53 neg_A54 neg_A55 neg_A56 neg_A57 neg_A58 neg_A59 neg_A60
